# Successful snare retrieval of a dislodged AVEIR leadless pacemaker from right pulmonary artery: a case report

**DOI:** 10.1186/s13019-026-04335-y

**Published:** 2026-06-14

**Authors:** Xuelian Song, Fan Wang, Feifei Zhang, Xiaoyong Qi, Yi Dang

**Affiliations:** 1 Department of Cardiology Center, Hebei General Hospital, Shijiazhuang, 050051 Hebei China; 2 Hebei Key Laboratory of Precision Medicine Translational Research on Cardiovascular Diseases, Shijiazhuang, China

**Keywords:** Leadless pacemaker, Complication, Dislodgment, Right pulmonary artery, Retrieval

## Abstract

**Background:**

Leadless pacemakers (LPs) are safer and more effective alternatives to traditional transvenous pacemakers, but LP detachment and displacement are serious complications.

**Case presentation:**

We report a case of AVEIR LP implantation, immediate dislodgement into the right pulmonary artery, and retrieval in an 82-year-old woman.

**Conclusion:**

The device was retrieved successfully using a gooseneck snare and the tri-loop snare of the retrieval catheter, then re-implanted.

## Introduction

Traditional pacemakers are associated with complications related to their leads and pacemaker pockets; common examples include pocket hematoma, pneumothorax or hemothorax, lead perforation or dislocation, lead fracture and insulation layer rupture, pocket rupture and pacing system infection, and venous thrombosis and obstruction [1]. Leadless pacemakers (LPs) overcome the shortcomings of traditional pacemakers, significantly reducing the total complication rate, and are increasingly widely used [2]. However, there is inevitably a learning curve for the mastery of the deployment of this new technology. We report a case of LP dislodgement into the pulmonary artery immediately after implantation in an 82-year-old woman. The device was retrieved successfully using gooseneck and triple-loop snares and re-implanted.

## Case report

An 82-year-old woman was admitted to the emergency room with severe symptoms of heart failure, including palpitations and shortness of breath after activity. The patient had a medical history of heart valve disease, heart failure, and interstitial lesions in both lungs. Holter monitoring showed atrial fibrillation and transient asystole (maximum R-R interval of 5.54 s, R-R interval > 3 s five times in 24 h). Transthoracic echocardiography (TTE) was performed on arrival and revealed a decreased ejection fraction, anterior mitral valve prolapse with massive regurgitation, and massive tricuspid valve regurgitation. Following pharmacological management, which improved the patient’s heart failure symptoms, the decision to implant a single-chamber LP was made based on Holter monitor data showing an R-R interval > 5 s and the presence of severe valvular heart disease. The procedure was performed after informed consent had been obtained from the patient.

### Procedure

**Fig. 1:**
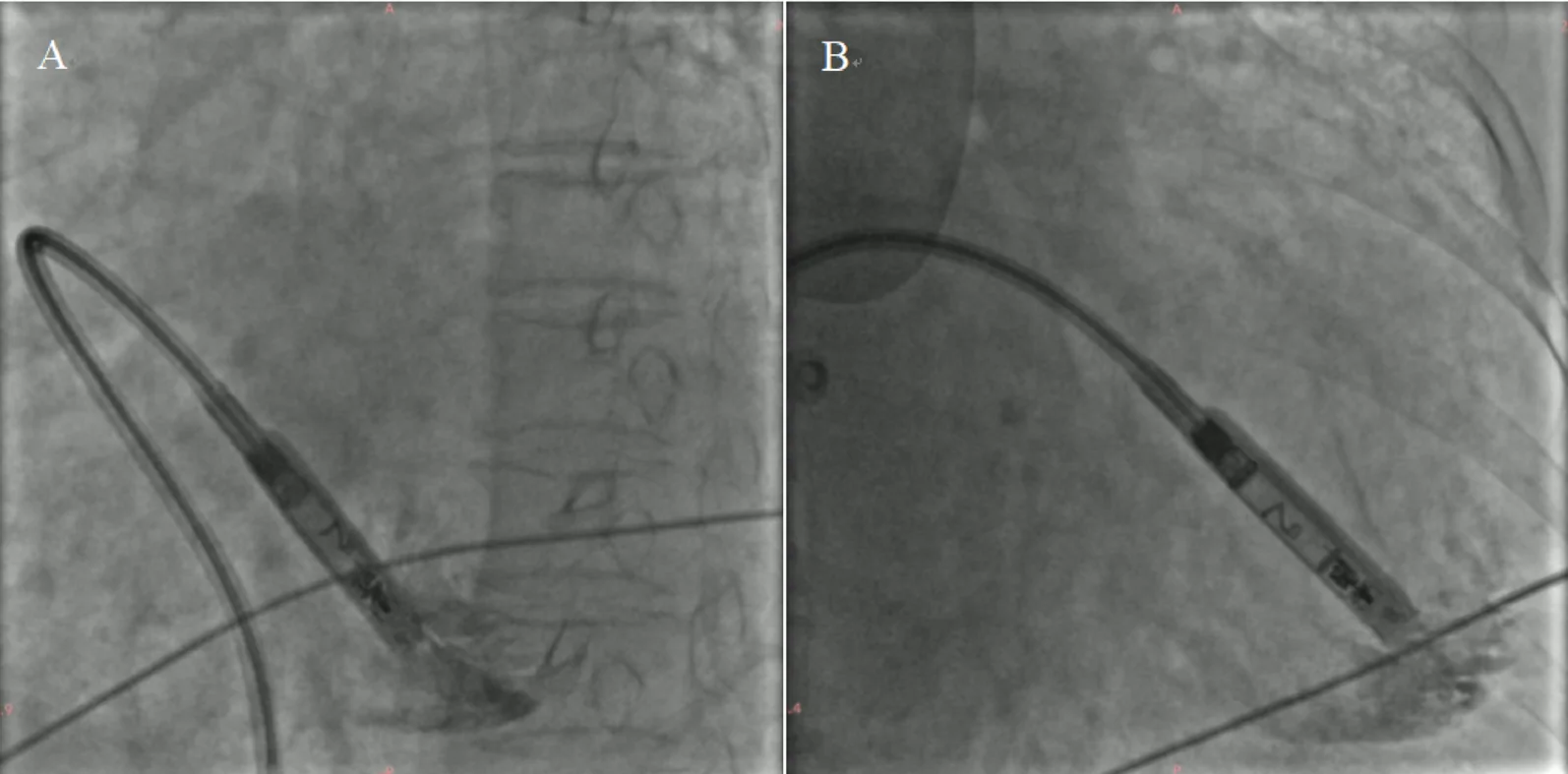
Fluoroscopic images of AVEIR leadless pacemaker implantation. Left (**A**) and right (**B**) anterior oblique contrast-enhanced images taken just before device fixation

**Fig. 2:**
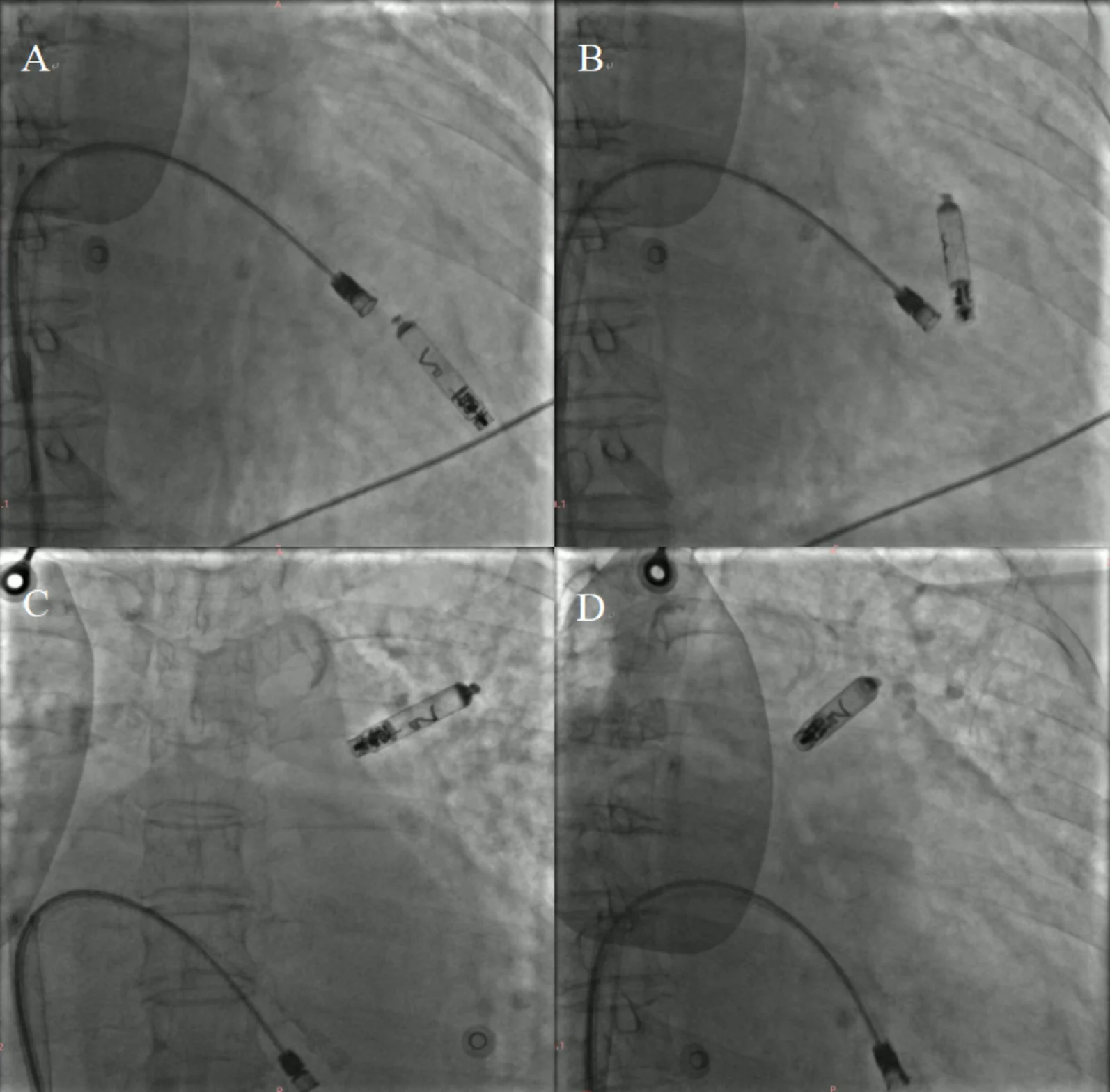
(**A**–**C**) The dislodgement sequence following AVEIR leadless pacemaker deployment. (**D**) Anteroposterior fluoroscopic image confirming device migration into the right pulmonary artery

Under local anesthesia induced with 2% lidocaine, the right femoral vein was punctured. A venous sheath was introduced, and venography confirmed the patency of the lower-extremity veins. A guidewire was advanced into the superior vena cava (SVC), followed by the insertion of a 6 F pigtail catheter into the right ventricle for right ventricular angiography. The catheter was then replaced with a stiff guidewire positioned in the SVC. After the administration of 2000 IU heparin, the femoral vein tract was pre-dilated using a dilator over the stiff guidewire. The delivery sheath assembly (with dilator) was subsequently introduced.The delivery catheter pre-loaded with the AVEIR LP (Abbott Medical Inc., Abbott Park, IL, USA) was advanced through this sheath. At the inferior vena cava (IVC)–right atrial junction, the LP was exposed, activated, and re-covered with the protective sleeve before being deployed to the low interventricular septum. Contrast injection confirmed the device’s appropriate final positioning (Fig. 1). Satisfactory mapping parameters were confirmed: the commanded electrogram showed ST-segment elevation, impedance of 380 Ω, a cardiac signal of 3.0 mV, and a pacing threshold of 1.25 V/0.4 ms.After 1.5 clockwise rotations into the myocardium under fluoroscopic guidance, a deflection test demonstrated secure fixation, and the pacemaker was deployed.Immediately upon deployment, however, the LP migrated to the right pulmonary artery, with obvious swinging movement of the docking button during cardiac contraction (Fig. 2). More critically, the device’s recapture feature was positioned in the distal right pulmonary artery, necessitating rotational repositioning for re-engagement.

**Fig. 3:**
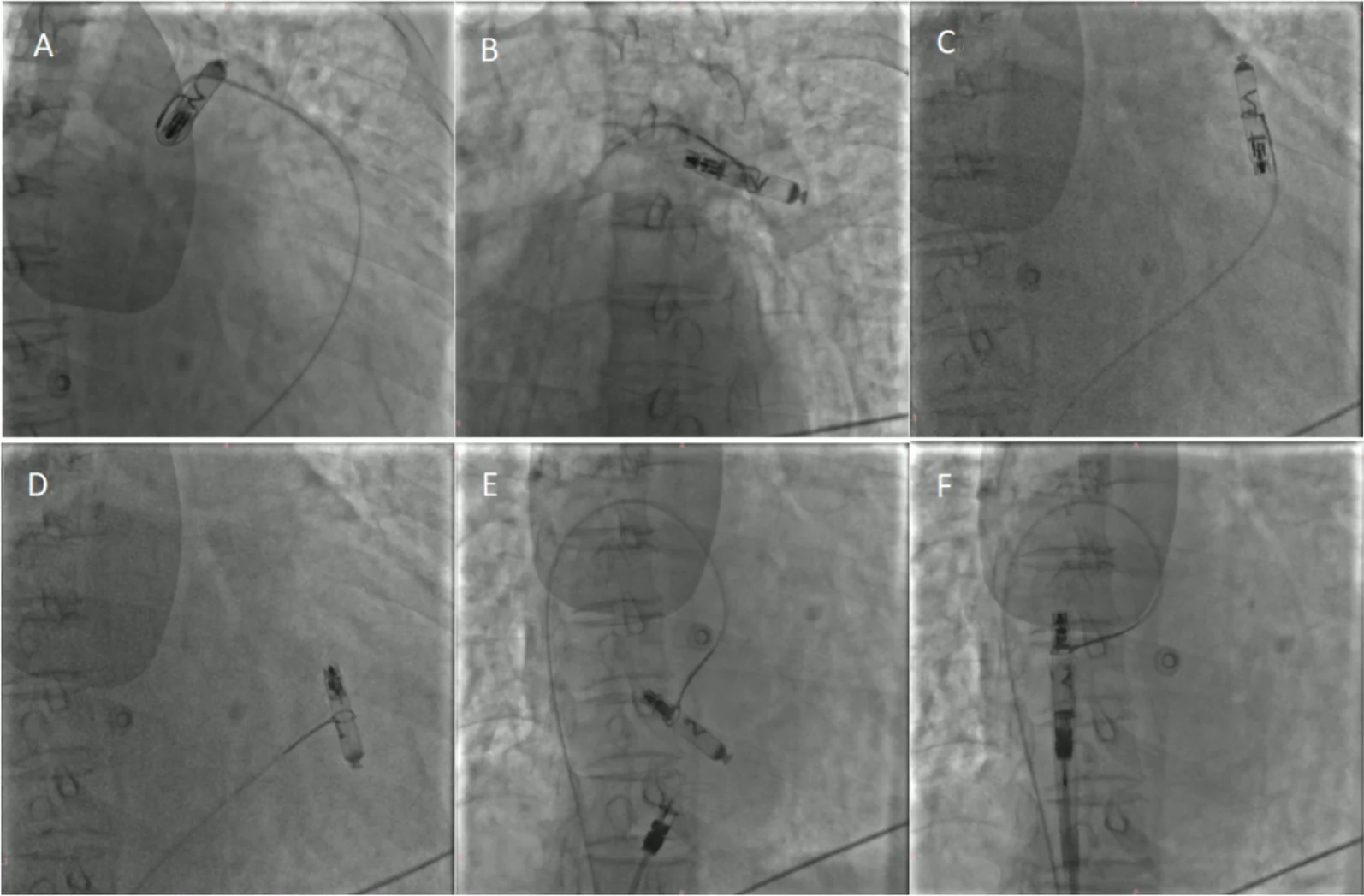
Fluoroscopic images obtained during AVEIR leadless pacemaker retrieval. (**A**) The gooseneck snare was placed around the device and (**B**) successfully grasped it. (**C**, **D**) The gooseneck snare was maneuvered into the inferior vena cava under traction, and was employed successfully for controlled reorientation of the pacemaker. (**E**, **F**) With the aid of the gooseneck snare, the tri-loop snare of the retrieval catheter was used to successfully grasp the docking button and dock the device

Retrieval of the dislodged device from the right pulmonary artery required traversing the tricuspid and pulmonary valves via the inferior vena cava (IVC), a maneuver for which bulky triple-loop snares are not ideally suited. We therefore employed a low-profile gooseneck snare (6 mm) for its superior navigability in the pulmonary artery.The gooseneck snare was advanced through a 7-Fr multipurpose catheter. Under LAO 45° fluoroscopy, the snare was looped around the distal tip of the device. With gentle traction, we successfully reoriented the device from a longitudinal to a transverse orientation, repositioning its docking button proximally, and maneuvered it back into the IVC.Once the device was stabilized in the IVC, the triple-loop snare of the retrieval catheter system was introduced to capture the main body. Using the deflection knob of the retrieval catheter, the catheter tip was steered to achieve fluoroscopic coaxial alignment, confirmed in both left anterior oblique (LAO) and right anterior oblique (RAO) projections. The snare was immediately cinched, and the device was successfully withdrawn into the retrieval sheath (Figs. 3).

Following confirmed engagement, the LP was redocked into the retrieval catheter’s docking cradle. We then repeated the implantation sequence. Satisfactory electrical parameters were confirmed during final device testing. Serial intraoperative and postoperative TTE readings demonstrated no evidence of pericardial effusion.The patient was informed about the intraprocedural complication and the successful retrieval/redeployment, and she expressed continued confidence in the procedure and gratitude for the avoidance of surgical extraction.

Post-procedural analysis indicated that the dislodgement was attributable primarily to inadequate myocardial fixation, as evidenced by suboptimal electrical parameters (a marginal increase in impedance from 290 Ω to 380 Ω). Following targeted medical therapy, the patient’s symptoms resolved and she was discharged. Assessments performed at 1-month and 1-year follow-up visits confirmed the maintenance of optimal LP parameters.

## Discussion

Current evidence indicates that cardiac effusion/perforation (0.13%), device dislodgement (0.13%), and sepsis (0.13%) are major complications occurring in the 30 days after LP placement; [3] however, most existing studies focus on the Medtronic Micra system((Medtronic, Minneapolis, MN, USA), [4] and a limited number of reports address Abbott AVEIR displacement and migration rates. The AVEIR LP, a new helix-fixation system, was designed to allow retrieval using a dedicated retrieval catheter. Unlike elective chronic retrieval of a well-fixed device, acute retrieval of a recently dislodged leadless pacemaker presents unique challenges. The device is often free-floating in a vascular structure (e.g., pulmonary artery) without significant encapsulation, making snare engagement difficult due to uncontrolled rotation. In our case, the double-snare technique was critical: the gooseneck snare allowed fine control to reorient the device’s docking button proximally, while the triple-loop snare provided a larger contact area to secure the body during withdrawal into the sheath.

In the case reported here, device dislodgement occurred immediately upon LP deployment, with subsequent migration to the right pulmonary artery. The mechanism of acute dislodgement in our case was likely multifactorial. Notably, the bloodpool impedance measured 290Ω immediately prior to deployment, which only rose to 380Ω after fixation—values considerably lower than the typical > 500Ω observed with successful myocardial anchoring. This suboptimal impedance trend suggests that the tines of the leadless pacemaker did not achieve sufficient penetration into the septal myocardium, possibly due to inadequate perpendicular orientation or excessive trabeculation at the deployment site. Therefore, we now advocate that confirmation of an adequate injury current and impedance rise (> 500Ω) should be mandatory before device release.When standard retrieval catheter access at the migration site proved to be infeasible, we utilized a gooseneck snare to capture and reorient the device. Final retrieval was accomplished in the right atrium using a triple-loop snare—a technique analogous to the double-snare “threading-a-needle” method described by Ip [5]. Minami et al. [6] identified tissue adherence at the proximal LP interface as a predictor of retrieval failure, with fibrotic encapsulation restricting device mobility during extraction, in our case, fluoroscopic imaging showed obvious swinging of the LP docking button during cardiac contraction without tissue adherence. Despite the absence of tricuspid valve injury in our case, the inherent risk of valvular damage during LP retrieval necessitates consideration of the use of advanced imaging modalities, specifically transesophageal or intracardiac echocardiography, to enhance procedural safety through the real-time detection of potential complications [7].

While several reports of Aveir embolization to the left pulmonary artery exist [8, 9], this is one of the few describing right pulmonary artery migration with this particular hybrid retrieval sequence.This case underscores the need for multimodal verification using imaging and electrophysiological parameters prior to LP release. Crucially, it also demonstrates that with appropriate techniques and operator expertise, dislodged AVEIR LPs can be recaptured, repositioned, and redeployed successfully. Our procedural approach contributes to the refinement of bailout strategies for LP systems.

## Conclusion

Several practical messages emerge from this case. First, operators should confirm an adequate rise in impedance or current of injury before final device release, as suboptimal electrical parameters may indicate inadequate fixation. Second, preparedness with multiple snare types—specifically a low-profile gooseneck snare for initial navigation and reorientation in the pulmonary artery, followed by a triple-loop snare for definitive capture—is essential when managing pulmonary artery embolization. Third, cinching of the snare should only be performed after confirming coaxial alignment under dual-projection fluoroscopy (left anterior oblique and right anterior oblique views) to ensure safe and effective retrieval.

### Limitations

Several limitations should be acknowledged. First, this is a single case report, which limits generalizability. Second, although 1-year follow-up data are available, longer-term outcomes beyond 12 months are not yet available. Third, successful retrieval cases are more likely to be reported, introducing potential publication bias toward favorable outcomes.

## Data Availability

No datasets were generated or analysed during the current study.
